# Supplementary material for: Predicting visual function by interpreting a neuronal wiring diagram
Source: Nature. 2024 Oct 2;634(8032):113–23. doi: 10.1038/s41586-024-07953-5 (PMC11446822; doi:10.1038/s41586-024-07953-5)
Supplement: Supplementary file 6 — CRF and ERF predictions for individual TmY4 and TmY9 cells. Analogous to Supplementary Data 3, but for TmY target types. Shown are the top four monosynaptic pathways, the strongest pathway passing through each of the top ten intermediary types (ranking from Extended Data Fig. 7), and the trisynaptic pathway Tm1–TmY–Dm3–TmY (see the section entitled Prediction of spatial normalization). [file 41586_2024_7953_MOESM6_ESM.zip › DataS4/TmY9qΓèÑ/720575940622166115.pdf]

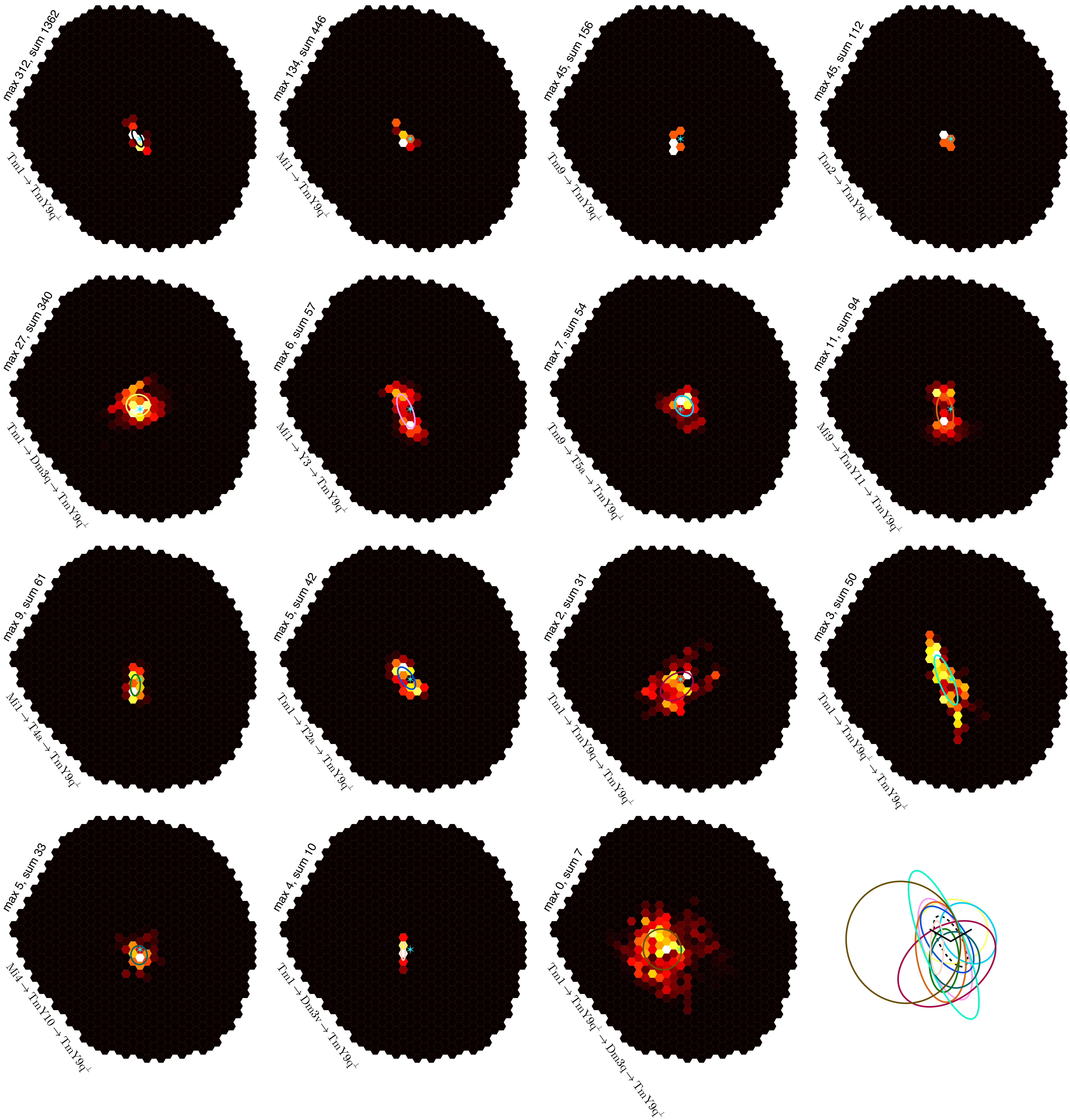
max 312, sum 1362
Tm1 → TmY9q⊥
max 134, sum 446
Mi1 → TmY9q⊥
max 45, sum 156
Tm9 → TmY9q⊥
max 45, sum 112
Tm2 → TmY9q⊥
max 27, sum 340
Tm1 → Dm3q → TmY9q⊥
max 6, sum 57
Mi1 → Y3 → TmY9q⊥
max 7, sum 54
Tm9 → T5a → TmY9q⊥
max 11, sum 94
Mi9 → TmY11 → TmY9q⊥
max 9, sum 61
Mi1 → T4a → TmY9q⊥
max 5, sum 42
Tm1 → T2a → TmY9q⊥
max 2, sum 31
Tm1 → TmY9q → TmY9q⊥
max 3, sum 50
Tm1 → TmY9q⊥ → TmY9q⊥
max 5, sum 33
Mi4 → TmY10 → TmY9q⊥
max 4, sum 10
Tm1 → Dm3v → TmY9q⊥
max 0, sum 7
Tm1 → TmY9q⊥ → Dm3q → TmY9q⊥
